# Outcome Measures Used to Assess Hand Activity in Amputee and Intact Populations: a Literature Review

**DOI:** 10.33137/cpoj.v5i2.39023

**Published:** 2022-12-25

**Authors:** K Carlyle, S Day

**Affiliations:** 1 Department of Biomedical Engineering, Faculty of Engineering, University of Strathclyde, Glasgow, United Kingdom.; 2 EPSRC Centre for Doctoral Training in Prosthetics and Orthotics, University of Salford, United Kingdom.

**Keywords:** Outcome Measures, Healthcare, Amputee, Upper Limb, Amputation, Rehabilitation, Prosthetics

## Abstract

**BACKGROUND:**

The human hand is critical in assisting with activities of daily living (ADL). Amputation of the hand can affect a person physically, socially and psychologically. Knowledge of outcome measures used to assess upper limb activity of intact and amputee populations may aid in guiding research to develop applicable measurement tools specific to the amputee population. Tools could aid developments in prosthetic design and prescription, which benefit both users and healthcare researchers.

**OBJECTIVE(S):**

This literature review examined outcome measurement tools used with non-amputee and amputee populations to assess hand activity. The objectives were to identify which characteristics of hand activity are captured by currently available measurement tools.

**METHODOLOGY:**

Searches were conducted using PubMed, Cochrane and ProQuest for studies investigating hand activity for amputee and non-amputee populations. A total of 15 studies were included. PRISMA guidelines were used to assist with study selection. Data extraction and narrative synthesis were carried out.

**FINDINGS:**

A total of 32 outcome measures were found. Frequently used tools were: Box and Block Test, Swedish Disabilities of the Arm Shoulder and Hand Questionnaire, and range of motion. Studies employed a combination of 2 to 12 tools. Themes extracted were: importance of function and quality of life, the need for realistic tasks, and the need for outcome measures specific of the population.

**CONCLUSION:**

There is a gap in research surrounding outcome measurement tools used to assess hand activity in the amputee population. A combination of outcome measures are required to obtain insight into the hand activities of intact and amputee populations. Function and quality of life are important aspects to consider when describing hand activity.

## INTRODUCTION

The hand is a complex structure responsible for powerful prehension and precise control. The range and adaptability achieved by the hand is due to the complex anatomical structure and precise control of movement and feedback by the central nervous system.^1^ Hands are capable of determining information by allowing identification of objects by their size, shape, surface, weight, texture, and thermal properties.^2^ Moreover, hands are crucial as terminal end-effectors in enabling people to carry out activities of daily living (ADL) and participate in society.^3^ They are vital for carrying out basic ADL such as feeding, dressing, and hygiene. Additionally, hands can be used as communication tools as demonstrated through the use of sign language and touch reading, and creative tools in music and dance.

If the hand is incapacitated due to trauma, tumour, infection, peripheral vascular disease, or congenital anomaly, then amputation may be the result. Roughly five to six thousand amputations are undertaken in the United Kingdom each year.^4^ Of these amputations, roughly one fifth are upper limb amputations.^5^ A solution to assist those with amputation in carrying out ADL lies in prosthetic devices. It has been noted that statistics relevant to the prevalence of limb amputation and prosthetic device provision are limited and often inconsistent.^6^ Studies have also shown that hand prostheses prescribed to users are not reaching their desired potential as users find them difficult to control and not fully functional.^6^ Despite advancements in prosthetic technology, people with amputations have significantly high rates of device rejection compared to other levels of prosthetic use.^7^ There are a range of devices and options available to people with amputation and their clinicians, however, more knowledge with respect to prosthetic performance may allow better prescription and design of prosthetics.

Recording patient and user outcomes is a recognised means of driving further research and development of technology.^8^ Outcome measurement tools have been used widely in research to assess limb activity. Such tools include quantitative functional tests and qualitative questionnaires. For health practitioners, standardised outcome measurement tools may allow them to better prescribe treatments for their patients.

This review contributes to healthcare research by reviewing the current state-of-the-art outcome measures used to assess hand activities. The review has an overarching aim of providing a basis for further research into outcome measurement tools for assessing real world use of upper limb prosthetics. The specific aim is to examine outcome measurement tools used to assess hand activities as no such review exists in the literature. The review achieves this by meeting the following objectives:

• Evaluating the range of existing outcome measurement tools for intact and amputee populations when assessing hand activity

• Examining and comparing various outcome measurement tools used for hand activities.

## METHODOLOGY

Reporting findings in a non-biased, usable format aids in advancing research and informing clinical decision making in the field of prosthetics and orthotics, and related fields. Since qualitative and quantitative tools are used to report outcome measures related to hand activity, a literature review was selected as a strong approach to synthesise such data and put it into context.^9^ Due to the different natures of the outcome measures expected to be found in the review, a narrative synthesis was performed to summarise data.^10^

**Figure 1:**
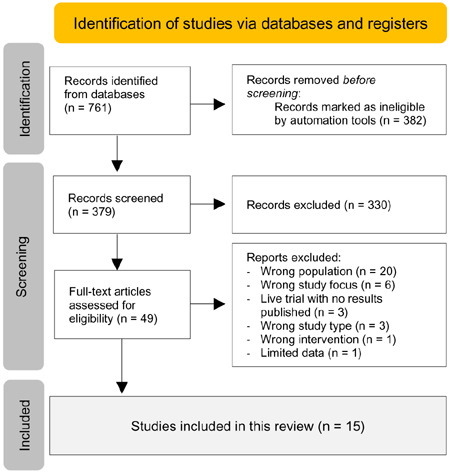
Study selection flowchart.

The literature review was conducted in accordance with PRISMA guidelines^11^ as shown in **Figure 1**. Searches were run in PubMed, Cochrane, ProQuest databases. Relevant keywords used in the search were (“outcome measures” OR “outcome measurement tool” OR “outcome measurement tools” OR “outcome measurement”) AND (“hand activity” OR “hand activities” OR “hand function”) AND (function* OR “quality of life” OR satisfaction) AND (amputee OR intact OR amputation) NOT (foot OR feet OR lower limb).

Results were filtered to meet the following inclusion criteria: written in English, including adult human participants, published between August 2009 and August 2021. Duplicates were removed. Study titles were screened and excluded if they reported on children, measurement tools that focus on arm movement (not hands), interventions not specific to population, feasibility studies and participants with neurological conditions without involvement of healthy controls.

The following types of study were excluded: systematic reviews, pilot studies, protocol developments, narrative reviews, feasibility studies and non-peer reviewed articles. Systematic reviews were excluded so the study only considered original research. Studies were included if they reported outcome measures and tools to assess adult human hand function and quality of life of unilateral upper limb amputees, bilateral upper limb amputees and the intact population.

Abstracts were screened from studies which passed or remained unclear following title screening. Full texts were obtained from studies which passed or remained unclear following screening. Full text screening was then conducted. The final studies were assessed for quality using a method derived from the CASP checklists.^12^ This method involved applying a set of questions to each study which took into account the validity of results, risk of bias, quality of data, ethical considerations and the applicability of results in the context of the research question and study population (**Table 1**).

**Table 1:** Quality appraisal checklist.

Author (year)	Sobuh et al., (2014)^13^	Lawrence et al., (2015)^14^	Resnik and Borgia (2016)^15^	Raveh et al., (2018)^16^	Lee et al., (2020)^17^	Eklund et al., (2009)^18^	Hruby et al., (2019)^19^	Resnik et al., (2020)^20^	Resnik et al., (2020)^21^	Speth et al., (2020)^22^	Wang et al., (2021)^23^	Hruby et al., (2017)^24^	de Boer et al., (2016)^25^	Bouma et al., (2018)^26^	Bernardon et al., (2015)^27^
Did the study address a clearly focused research question?	Y	Y	Y	Y	Y	Y	Y	Y	Y	Y	Y	Y	Y	Y	Y
Was the recruitment strategy appropriate to the aims?	Y	Y	Y	Y	Y	Y	Y	Y	Y	Y	Y	Y	Y	Y	Y
Are there any conflicts of interest?	N	N	N	N	N	N	N	Y (declared financial grant)	N	Y (declared financial grant)	N	N	N	N	N
Was there a clearly defined study protocol?	Y	Y	Y	Y	Y	Y	Y	Y	Y	Y	Y	Y	Y	Y	Y
Do the benefits of the experimental intervention outweigh the harms and costs?	Y	Y	Y	Y	Y	Y	Y	Y	Y	Y	Y	Y	Y	Y	Y
Have ethical issues been taken into consideration?	Y	Y	Y	Y	Y	Y	Y	Y	Y	Y	Y	Y	Y	Y	Y
Is there a clear statement of findings?	Y	Y	Y	Y	Y	Y	Y	Y	Y	Y	Y	Y	Y	Y	Y
Can the results be applied to the context of hand and upper limb activity?	Y	Y	Y	Y	Y	Y	Y	Y	Y	Y	Y	Y	Y	Y	Y
Does the study find anything new or useful?	Y	Y	Y	Y	Y	Y	Y	Y	Y	Y	Y	Y	Y	Y	Y

Ethical approval was not required to complete this review as no subject participation or handling of sensitive information were carried out. Ethics surrounding the studies included in this review were considered. All studies involving subjects and confidential information were checked to ensure quality data was collected with appropriate ethical approval.

### Data Analysis

All outcome measures used in each study were recorded and the frequency of use of each outcome measure was noted. Modified or adapted versions of tools - such as the original DASH and QuickDASH – were grouped together. Measures which were similar in nature through describing the same variables were grouped together. Strength variables such as grip strength and precision force were grouped together to represent force control (FC). Joint angle measures were grouped together (ROM). Shape texture identification test and self-rating of tactile gnosis were grouped together (tactile gnosis/TG). Upper extremity functional scale and patient specific functional scales were grouped together (functional scales/FS). Duration of hand movements and task movement times were grouped together (temporal measures/TM). Outcome measures were also grouped into the following types: ‘functional’, ‘quality of life (QoL)’ and ‘functional and QoL’ measures. Narrative synthesis was carried out with the aid of thematic analysis to determine themes.

## RESULTS

Following searching and screening, 15 studies were reviewed (**Table 2**).

**Table 2:** List of included papers

**Authors**	**Title**	**Year**
Sobuh et al.,^13^	Visuomotor behaviours when using a myoelectric prosthesis	2014
Lawrence et al.,^14^	Outcome measures for hand function naturally reveal three latent domains in older adults: strength, coordinated upper extremity function, and sensorimotor processing	2015
Resnik and Borgia^15^	Responsiveness of outcome measures for upper limb prosthetic rehabilitation	2016
Raveh et al.,^16^	Myoelectric prosthesis users improve performance time and accuracy using vibrotactile feedback when visual feedback is disturbed	2018
Lee et al.,^17^	Clip-On IMU system for assessing age-related changes in hand functions	2020
Eklund et al.,^18^	Hand function and disability of the arm, shoulder and hand in Charcot-Marie-Tooth disease	2009
Hruby et al.,^19^	Bionic upper limb reconstruction: a valuable alternative in global brachial plexus avulsion injuries-a case series	2019
Resnik et al.,^20^	A national survey of prosthesis use in veterans with major upper limb amputation: comparisons by gender	2020
Resnik et al.,^21^	Function and quality of life of unilateral major upper limb amputees: effect of prosthesis use and type	2020
Speth et al.,^22^	Assessment of tree-based statistical learning to estimate optimal personalized treatment decision rules for traumatic finger amputations	2020
Wang et al.,^23^	Application of machine learning to the identification of joint degrees of freedom involved in abnormal movement during upper limb prosthesis use	2021
Hruby et al.,^24^	Algorithm for bionic hand reconstruction in patients with global brachial plexopathies	2017
de Boer et al.,^25^	Intermanual transfer effects in below-elbow myoelectric prosthesis users	2016
Bouma et al.,^26^	Musculoskeletal complaints in individuals with finger or partial hand amputations in the Netherlands: a cross-sectional study	2018
Bernardon et al.,^27^	Bilateral hand transplantation: functional benefits assessment in five patients with a mean follow-up of 7.6 years (range 413 years)	2015

A data extraction table (**Table 3**) was used to record number of outcome measures, types of outcome measures, participant demographics and ethical considerations.

**Table 3:** Data extraction table.

**Authors/year**	**Title**	**Ethical Concerns?**	**Type of Study**	**Participant Demographics**	**Number of Participants**	**Intact, amputee, prosthesis user or combination**	**Setting**	**Self-reported, clinician observed or combination?**
Sobuh et al., 2014 ^13^	Visuomotor behaviours when using a myoelectric prosthesis	No	quantitative	intact adults and adult myoelectric prosthesis users	11	combination (intact and prosthesis user)	university	clinician observed
Lawrence et al., 2015 ^14^	Outcome measures for hand function naturally reveal three latent domains in older adults: strength, coordinated upper extremity function, and sensorimotor processing	No	quantitative	healthy older adults and older adults with osteoarthritis of the CMC joint	99	intact	university/rehab centre	clinician observed
Resnik and Borgia, 2016 ^15^	Responsiveness of outcome measures for upper limb prosthetic rehabilitation	No	quasi-experiment al study	adult upper limb amputees	39	amputee	veterans affairs sites	combination
Raveh et al., 2018 ^16^	Myoelectric prosthesis users improve performance time and accuracy using vibrotactile feedback when visual feedback is disturbed	No	quantitative	transradial amputees using a myoelectric prosthesis with normal or corrected eyesight	12	prosthesis user	laboratory	combination
Lee et al., 2020 ^17^	Clip-On IMU system for assessing age-related changes in hand functions	No	quantitative	healthy adults (aged 20-31 and 75-89)	34	intact	university	clinician observed
Eklund et al., 2009 ^18^	Hand function and disability of the arm, shoulder and hand in charcot-marie-tooth disease	No	quantitative	adults with charcot marie tooth and healthy controls	60	intact	hospitals/clinics	combination
Hruby et al., 2019 ^19^	Bionic upper limb reconstruction: a valuable alternative in global brachial plexus avulsion injuries-a case series	No	quantitative	adults with complete bracial plexus injury who underwent bionic reconstruction after high level upper limb amputation	5	prosthesis user	university	combination
Resnik et al., 2020 ^20^	A national survey of prosthesis use in veterans with major upper limb amputation: comparisons by gender	No	cross-sectional survey	adult veterans with upper limb amputation who had been treated between 2010 and 2015	808	amputee	telephone survey	self-reported
Resnik et al., 2020 ^21^	Function and quality of life of unilateral major upper limb amputees: effect of prosthesis use and type	No	cross-sectional survey	adult veterans with unilateral upper limb amputation who had been treated between 2010 and 2015	755	amputee	telephone survey	self-reported
Speth et al., 2020 ^22^	Assessment of tree-based statistical learning to estimate optimal personalized treatment decision rules for traumatic finger amputations	No	cohort study	adult patients who underwent revision amputation or replantation	185	amputee	various research centres	combination
Wang et al., 2021 ^23^	Application of machine learning to the identification of joint degrees of freedom involved in abnormal movement during upper limb prosthesis use	No	quantitative	adults with no upper limb disability, trained on deka bypass or body powered bypass	24	intact	laboratory	clinician observed
Hruby et al., 2017 ^24^	Algorithm for bionic hand reconstruction in patients with global brachial plexopathies	No	quantitative	adults with posttraumatic global brachial plexopathies	5	prosthesis user	university	combination
de Boer et al., 2016 ^25^	Intermanual transfer effects in below-elbow myoelectric prosthesis users	No	case-control study	myoelectric prosthesis users and controls	44	combination (intact and prosthesis user)	university/rehab centre	clinician observed
Bouma et al., 2018 ^26^	Musculoskeletal complaints in individuals with finger or partial hand amputations in the Netherlands: a cross-sectional study	No	cross-sectional study	adult finger and hand amputees and healthy controls	201	combination (intact and amputee)	questionnaire sent to participants	self reported
Bernardon et al., 2015 ^27^	Bilateral hand transplantation: functional benefits assessment in five patients with a mean follow-up of 7.6 years	No	quantitative	adults who underwent hand and forearm allotransplantation following trauma	5	intact (post transplantation)	rehabilitation clinic	combination

Studies were published between the years 2009 and 2021. Across the studies, participants were categorised in the following populations: able bodied, able bodied using upper limb bypass equipment, upper limb prosthesis users; people with osteoarthritis, Charcot-Marie-Tooth disease, upper limb/finger/hand amputation and recipients of hand and forearm allotransplantation. Studies were conducted using intact only, intact and amputee/prosthesis user, and amputee/prosthesis user only populations. Studies involving participants with osteoarthritis and Charcot-Marie-Tooth used healthy controls, enabling their inclusion within the review.

A total of 32 outcome measures were identified within the studies reviewed (**Table 4**).

**Table 4:** List of outcome measures, acronyms, type and description of measures.

**Outcome Measure**	**Acronym**	**Type**	**Description**
Box and Block test^28^	BBT	Functional	Test of manual dexterity
Swedish Disabilities of the Arm Shoulder and Hand Questionnaire^29^	DASH	Functional & QoL	Self-reported measure of disability and symptoms in relation to the upper limb
Range of Motion	ROM	Functional	Measure of level of movement achieved
Force Control	FC	Functional	Measure of strength achieved
Southampton Hand Assessment Procedure^30^	SHAP	Functional	Test of hand function using abstract objects and ADL
Orthotics and Prosthetics User’s Survey^31^	OPUS	Functional & QoL	Self-reported survey of outcomes and satisfaction with orthotic/prosthetic devices and services
Tactile Gnosis	TG	Functional	Ability to detect information through touch
9 Hole Peg test^32^	9HPT	Functional	Measure of finger dexterity
36-Item Short Form Survey^33^	SF-36	QoL	Self-reported measure of quality of life
Temporal measures	TM	Functional	Time-based activities and tasks
Edinburgh Handedness Inventory^34^	EHI	Functional	Assessment of hand dominance
Modified Action Research Arm test^35^	mARAT	Functional	Assessment of hand function during grasp, pinch, grip and gross movements
Visual Analogue Scale^36^	VAS	QoL	Measure of severity of symptoms
Trinity Amputation and Prosthetic Experience Scale^37^	TAPES	QoL	Self-reported measure of experiences and satisfaction with regards to acquired amputation and prosthesis adjustment
Veterans SF-12 Health Survey with physical and mental components^38^	VR-12	Functional & QoL	Self-reported measure of health
Jebsen-Taylor Hand Function tests^39^	JTHFT	Functional	Test of hand function carrying out a range of different ADL-based tasks
Functional Scales	FS	Functional	Measure of function
Pain	Pain	QoL	Assessment of patient/user pain
Activity measure for upper limb amputation^40^	AM-ULA	Functional	Measure of upper limb activity performance
Michigan Hand Outcomes Questionnaire^41^	MHQ	Functional & QoL	Self-reported measure of hand outcomes including pain, function, aesthetics, ADL, work and satisfaction
Gaze Behaviour	GB	Functional	Assessing visual responses during tasks
University of New Brunswick Skill and Spontaneity tests^42^	UNB-SST	Functional	Non-timed measure of function
Prevalence of Musculoskeletal Complaints	MSC	QoL	Self-reported indication of physical symptoms
Upper extremity work demands score	UEWD	Functional & QoL	Measure specifically related to work tasks
Patient-reported work productivity	PR-WP	QoL	Self-reported indication of level of ability to participate in work
Modified Kapandji Index^43^	mKI	Functional	Measure of hand mobility
Carroll Upper Extremity Function test^44^	UEFT	Functional	Measure of functional impairment and severity
Purdue Pegboard test^45^	PPT	Functional	Measure of gross upper limb movement and finger dexterity
Self-subjective global evaluations	SSGE	QoL	Self-reported measure of quality of life
400 Point Assessment^46^	400-PA	Functional	Test of function in tasks, strength, mobility and handling
Direct observation by therapist while performing tasks	DTO	Functional	Clinician-researcher observing participant without using a specific measure
Hand Transplantation Score System^47^	HTSS	Functional & QoL	Measure of ability and quality of life following hand transplantation

**Figure 2:**
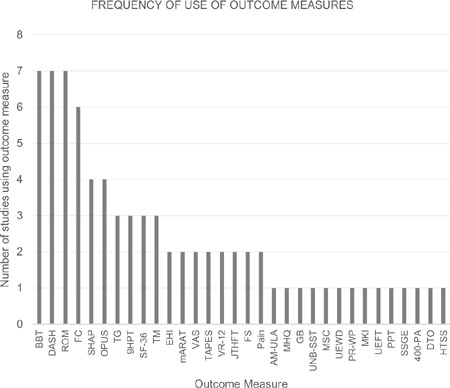
Graph depicting number of times each outcome measure was used across the included studies.

The most frequently used tools and measures, as shown in **Figure 2**, were the BBT (7), DASH (7), ROM (7) and FC (6). All studies used a combination of measures, ranging from 2 to 12 outcome measures assessed per study.

**Figure 3:**
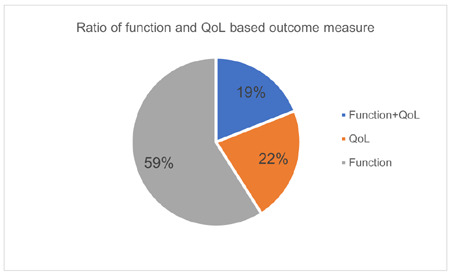
Pie chart showing that most outcome measures assess function, with a smaller proportion assessing QoL.

The majority (59%) of outcome measures listed in **Table 4** are functional based measures. While function-based outcome measures were more prevalent within studies, some outcome measurement tools (19%) assess both function and quality of life (**Figure 3**).

The following quotes of relevance to the research topic were extracted from included studies:

• *“the most objective of the commonly used upper limb evaluation tools are based on time to perform a structured set of tasks, but use of these in isolation gives limited insight into ease of use of a prosthesis”*^13^

• *“the central question here is, what should we use to quantify hand function considering that we have so many choices of assessment tools and even more outcome measures stemming from these tools?”*^14^

• *“leaders in upper limb prosthetic rehabilitation understand the importance of choosing outcome measures that have been evaluated for persons with upper limb amputation and that are reliable, valid, and responsive to change”*^18^

• *“another important aspect in prosthetic rehabilitation research is the use of valid outcome measures”*^19^

The following themes were derived from the papers in relation to assessing hand activity: the importance of function and quality of life, the need for realistic tasks, and the need for outcome measures specific to the population.

## DISCUSSION

A The review depicts a range of outcome measures used to measure hand activity in amputee and intact populations. There was a clear indication that use of multiple outcome measures is optimum to evaluate hand activity. It could be true that it is difficult to select one tool due to lack of tools which bring together various aspects of hand activity such as function, pain, and satisfaction.

The reason behind choice of tool may be due to location of the study – for example in-clinic or telephone surveys. Additionally, choice of tool may be a consequence of the fact that not all tools are validated for use in amputee populations. It is possible that researchers select tools based on personal preference rather than appropriateness. Tools involving participant-reporting of data may not reflect physical performance, thus may not be sufficient. Nonetheless, self-reported studies, which occur as surveys or interviews can attract larger sample sizes. No specific guidelines related to hand activities or therapy were highlighted within the included studies, emphasising the need for their development. There was a lack of diverse devices included in the studies with most studies involving prostheses featuring only myoelectric devices. Finally, there is limited information on outcome measures for cosmetic devices.

### Theme 1: The importance of function and quality of life when assessing hand activity

A key theme was the value of both functional and quality of life assessments of hand activity. Despite this finding, 59% of outcome measures used were functional measures and only 22% quality of life measures. By using a combination of outcome measurement tools, the researchers were frequently able to capture functional, and quality of life data. However, there is no standard combination, or designed pairing of tools to obtain all information. This highlights the need for development of guidelines that are comparative of both contexts.

While functional domains and tasks were considered repeatedly throughout studies, it is evident that these variables affect quality of life. These variables can affect ability to participate in work and social environments which subsequently attributes to an improved quality of life.

Pain was another common theme found throughout studies. Pain should be considered throughout performance of various functional tasks as such tasks may conversely impair function. Presence of pain is likely to have a negative effect on quality of life. It is important to involve the participants’ self-reported outcomes of parameters such as pain to create a picture of their overall satisfaction. Nonetheless, self-reported outcomes are limited due to self-reporting bias.

Outcome measures, which account for functional failures, may be advantageous in the amputee and prosthetic user population to account for actions such as failed prehension. This could provide key information about device performance as well as participant function.

### Theme 2: Need for realistic tasks when assessing hand activity

Another major theme was the importance of ADL. Many of the functional outcome measures were used in combination with other tools to put function into context. Most of the studies investigating hand function, and the outcome measures included were structured in that the participants were given precise instructions to complete tasks. It must be noted that the ability to place pegs in a board, as required within the 9HPT and PPT, does not correlate to information about key parameters such as strength or function. Similarly this process does not correlate with the skill required to carry out typical ADL. Likewise, the BBT was one of the most frequently used measures and is simple to set up - but transferring blocks between compartments is one repetitive process which is not likely implemented in most ADL.

An overlooked element is participant-led activities during assessment. In prosthetic user populations, it would be pragmatic to ask the participant to complete tasks with their prosthesis on and off to account for a range of scenarios. It should not be assumed that users wear their device consistently. Studies should include both unilateral and bilateral tasks to represent a wider range of real-life scenarios. In addition, most studies were carried out in research laboratories, clinics, and universities. This means the researcher has control within the study and this weakens the link between the hand activities and real-world applications.

Shifting from basic tasks to real world object manipulation, such as turning keys or picking up a mug, would provide an extra dimension in relating clinical studies to applications in the real world.

### Theme 3: Need for outcome measures specific to population

Different populations are known to have different key components of hand function, so the focus of functional assessment tools should be tailored to the prosthesis user population. It was also highlighted within the reviews that people value parameters differently, for example dexterity may be of more importance than strength. It would be advantageous to understand the parameters which are of importance to prosthesis users before using tools to assess hand activity. Performance-based outcome measures used to assess hand activity often compare the performance of an amputee or prosthesis user with the intact population. This is not an optimal comparison since the populations have various levels of function and goals. Low level strength is required to facilitate ADL and functional tasks, so people with amputation who may have a level of strength too low to carry out such tasks may be unable to be assessed with such tools. Also, there is a level of dexterity required for tools such as tactile gnosis assessments. If outcome measurement tools can only be used in populations with a baseline level of ability, then tools must be adapted or developed to involve people who do not satisfy this criterion. Further, many of the tools used are temporal based and therefore do not give indications into ease of use, which is an important parameter when investigating the amputee and prosthesis user populations. Another argument for using outcome measures specific to the population is that a specific measurement may alleviate the need for recruiting healthy, intact participants when researchers are interested in outcomes of amputee or prosthesis users only.

### Limitations

A limitation of this review is that data from pilot studies, reviews and protocol development studies were not included. Therefore, outcome measures considered are not a comprehensive list. Inclusion was limited to studies published in more recent years to ensure conclusions were reflective of the current state of technology and practice. Studies which only included participants with neurological conditions were excluded to ensure the correct population was analysed. Studies which used stroke-specific tools were excluded as outcome measurement tool selection by the research may be biased towards the stroke population and thus not a true reflection of amputee or intact populations. Small sample sizes within many included studies are another limiting factor, as this restricts data available for assessment. Studies with larger sample sizes were self-reported surveys which are limited in terms of outcome measurement tools which can be used. Only one reviewer screened and selected articles for inclusion, thus presenting a potential element of bias in terms of study selection.

## CONCLUSION

There is a definite gap in research surrounding outcome measurement tools used to assess hand activity in the amputee and prosthesis user population. Results from this review of outcome measures used in amputee and intact populations, showed that a combination of outcome measures are currently used to obtain insight into hand activities of intact and amputee populations. There are no set guidelines or recommended pairings of tools, and key information about hand activity could potentially be missed during investigations.

The reason a combination of tools are used is partially because currently used outcome measurement tools are limited for use in amputee and prosthesis user populations. Some measurement tools were not validated for upper limb amputees, and some involving temporal based tasks may not have been appropriate for assessing function in prosthesis users. Additionally, combinations of tools are used because both function and quality of life measurements are deemed important. These aspects are of particular interest in amputee and prosthetic user populations where factors such as pain, social participation and satisfaction are of high importance to both the person themselves, healthcare practitioners and prosthesis developers.

Tools should be developed with both functional and quality of life measurements taken into consideration as well as tasks which pose a likeness to carrying out ADL. Finally, tools should be developed specific to the population to ensure that measurements are valid, useful, and specific.

## DECLARATION OF CONFLICTING INTERESTS

The Authors declare that there is no conflict of interest.

## AUTHORS CONTRIBUTION

**Kirsty Carlyle**: Designed the research question and study design, conducted the literature search and study selection, writing the manuscript.

**Sarah Day**: Designed the research question and study design, writing the manuscript.

## SOURCES OF SUPPORT

This work was supported by the UK Engineering and Physical Sciences Research Council (EPSRC) grant EP/S02249X/1 for the Centre for Doctoral Training in Prosthetics and Orthotics.

## ETHICAL APPROVAL

Ethical approval was not needed.
